# 2,4-Dioxo-1-(prop-2-yn­yl)-1,2,3,4-tetra­hydro­pyrimidine-5-carbaldehyde

**DOI:** 10.1107/S1600536811032272

**Published:** 2011-08-17

**Authors:** Yan He, Liang-Yan Cui, Xin-Ying Zhang

**Affiliations:** a School of Chemistry and Environmental Science, Henan Key Laboratory for Environmental Pollution Control, Henan Normal University, Xinxiang, Henan 453007, People’s Republic of China

## Abstract

In the crystal structure of the title compound, C_8_H_6_N_2_O_3_, the mol­ecules are linked by a pairs of inter­molecular N—H⋯O hydrogen bonds, forming inversion dimers. The aldehyde group is in the same plane as the pyrimidine ring [with a maximum deviation of 0.083 (2) Å for the O atom), and the linear propargyl group [C—C—C = 178.99 (19)°] makes a dihedral angle of 74.36 (13)° with the ring.

## Related literature

For applications of acyclic pyrimidine nucleosides, see: De Clercq (2009 ▶, 2010*a* ▶,*b* ▶); Fan *et al.* (2011 ▶).
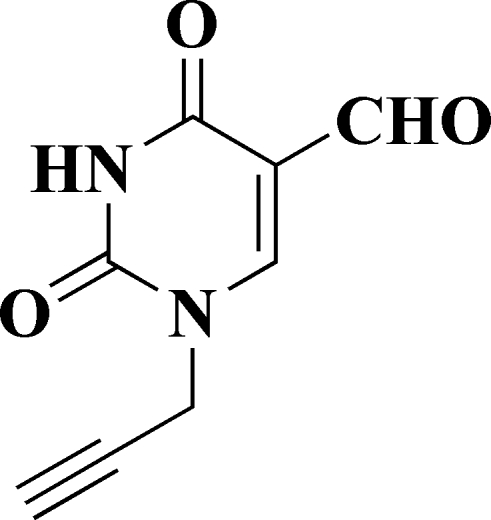


## Experimental

### 

#### Crystal data

C_8_H_6_N_2_O_3_
*M* *_r_* = 178.15
Monoclinic, 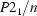

*a* = 5.1756 (7) Å
*b* = 8.4877 (12) Å
*c* = 18.565 (3) Å
β = 90.611 (2)°
*V* = 815.5 (2) Å^3^
*Z* = 4
Mo *K*α radiation
μ = 0.11 mm^−1^
*T* = 296 K
0.41 × 0.37 × 0.25 mm

#### Data collection

Bruker SMART CCD area-detector diffractometer
Absorption correction: multi-scan (*SADABS*; Bruker, 1997 ▶) *T*_min_ = 0.955, *T*_max_ = 0.972
5826 measured reflections
1520 independent reflections
1261 reflections with *I* > 2σ(*I*)
*R*_int_ = 0.020

#### Refinement

*R*[*F* ^2^ > 2σ(*F* ^2^)] = 0.040
*wR*(*F* ^2^) = 0.123
*S* = 1.08
1520 reflections
118 parameters
H-atom parameters constrained
Δρ_max_ = 0.14 e Å^−3^
Δρ_min_ = −0.23 e Å^−3^

### 

Data collection: *SMART* (Bruker, 1997 ▶); cell refinement: *SAINT* (Bruker, 1997 ▶); data reduction: *SAINT*; program(s) used to solve structure: *SHELXS97* (Sheldrick, 2008 ▶); program(s) used to refine structure: *SHELXL97* (Sheldrick, 2008 ▶); molecular graphics: *SHELXTL* (Sheldrick, 2008 ▶); software used to prepare material for publication: *SHELXTL*.

## Supplementary Material

Crystal structure: contains datablock(s) I, global. DOI: 10.1107/S1600536811032272/is2760sup1.cif

Structure factors: contains datablock(s) I. DOI: 10.1107/S1600536811032272/is2760Isup2.hkl

Supplementary material file. DOI: 10.1107/S1600536811032272/is2760Isup3.cml

Additional supplementary materials:  crystallographic information; 3D view; checkCIF report

## Figures and Tables

**Table 1:** Hydrogen-bond geometry (Å, °)

*D*—H⋯*A*	*D*—H	H⋯*A*	*D*⋯*A*	*D*—H⋯*A*
N2—H2⋯O1^i^	0.86	1.98	2.8329 (18)	174

Symmetry code: (i) 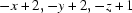.

**Fig. 1.**
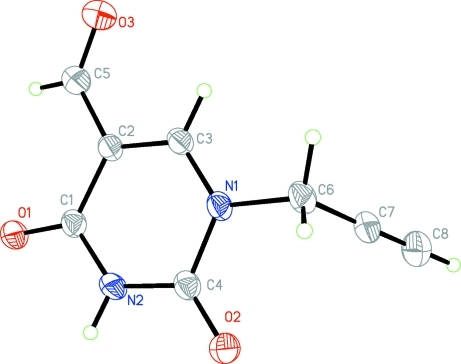
Molecular structure of the title compound, with displacement ellipsoids drawn at the 30% probability level.

**Fig. 2.**
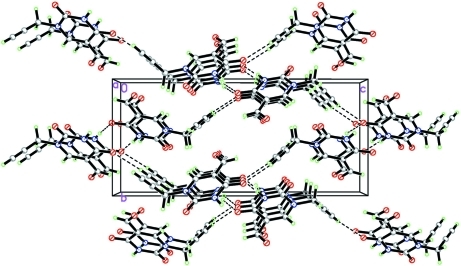
Crystal packing of the title compound with view along the a axis. Intermolecular N—H···O hydrogen bonds are shown as dashed lines.

## supplementary crystallographic information

### Comment

Acyclic pyrimidine nucleosides have drawn much attention because of their insteresting structures and broad utilizations as effective drugs for the treatment of diseases caused by herpes simplex virus (HSV) and varizella zoster (VZV) (De Clercq, 2009, 2010*a*,*b*). The title compound can be used as a powerful synthon for the preparation of acyclic pyrimidine nucleoside derivatives with potential biological activities due to the rich and extensive chemistry of the aldehyde carbonyl (Fan, 2011). Herein, we report the synthesis and crystal structure of the title compound.

In the title compound, C_8_H_6_N_2_O_3_, all the atoms in the pyrimidine ring, atoms connected directly with the pyrimidine ring and atoms in the aldehyde carbonyl group in the 5-position of the pyrimidine ring are in the same plane, which means there is a big conjugated system in the molecule. The linear structure of the propynyl group is connected with the big plane at an angle of 150.3°. In the crystal structure, the molecules are linked *via* intermolecular N—H···O hydrogen bond.

### Experimental

To a solution of K_2_S_2_O_8_ (16.5 mmol) and CuSO_4_ (3.2 mmol) in 30 ml H_2_O was added a CH_3_CN solution (25 ml) of 5-methyl-1-(prop-2-ynyl)pyrimidine-2,4(1*H*,3*H*)-dione (8 mmol) and 2,6-lutidine (3.2 ml). The mixture was stirred at 60 °C for 5 h. Upon completion, the mixture was concentrated to half of the initial volume, and the remaining solution was extracted with EtOAc. The organic layer was washed with H_2_O. The aqueous layers were combined and back-extracted with CHCl_3_. Then the organic layers were combined, dried over Na_2_SO_4_, and then concentrated. The residue was purified through silica gel column chromatography with a mixture of methylene chloride-methanol (60:1, *v*/*v*) as eluent to give 1,2,3,4-tetrahydro-2,4-dioxo-1-(prop-2-ynyl)- pyrimidine-5-carbaldehyde. Single crystals of the title compound were obtained by slow evaporation of the solvent from a methylene chloride-methanol (60:1 *v*/*v*) solution.

### Refinement

H atoms were positioned geometrically and refined using riding model, with C—H = 0.93 or 0.97 Å, and N—H = 0.86 Å, and with *U*_iso_(H) = 1.2*U*_eq_(C, N).

### Crystal data

**Table:**

C_8_H_6_N_2_O_3_	*F*(000) = 368
*M**_r_* = 178.15	*D*_x_ = 1.451 Mg m^−^^3^
Monoclinic, *P*2_1_/*n*	Mo *K*α radiation, λ = 0.71073 Å
Hall symbol: -P 2yn	Cell parameters from 2188 reflections
*a* = 5.1756 (7) Å	θ = 2.6–26.7°
*b* = 8.4877 (12) Å	µ = 0.11 mm^−^^1^
*c* = 18.565 (3) Å	*T* = 296 K
β = 90.611 (2)°	Block, colourless
*V* = 815.5 (2) Å^3^	0.41 × 0.37 × 0.25 mm
*Z* = 4	

### Data collection

**Table:**

Bruker SMART CCD area-detector diffractometer	1520 independent reflections
Radiation source: fine-focus sealed tube	1261 reflections with *I* > 2σ(*I*)
graphite	*R*_int_ = 0.020
phi and ω scans	θ_max_ = 25.5°, θ_min_ = 2.6°
Absorption correction: multi-scan (*SADABS*; Bruker, 1997)	*h* = −6→6
*T*_min_ = 0.955, *T*_max_ = 0.972	*k* = −10→10
5826 measured reflections	*l* = −22→21

### Refinement

**Table:**

Refinement on *F*^2^	Primary atom site location: structure-invariant direct methods
Least-squares matrix: full	Secondary atom site location: difference Fourier map
*R*[*F*^2^ > 2σ(*F*^2^)] = 0.040	Hydrogen site location: inferred from neighbouring sites
*wR*(*F*^2^) = 0.123	H-atom parameters constrained
*S* = 1.08	*w* = 1/[σ^2^(*F*_o_^2^) + (0.0694*P*)^2^ + 0.1695*P*] where *P* = (*F*_o_^2^ + 2*F*_c_^2^)/3
1520 reflections	(Δ/σ)_max_ < 0.001
118 parameters	Δρ_max_ = 0.14 e Å^−^^3^
0 restraints	Δρ_min_ = −0.23 e Å^−^^3^

### Special details

**Table:**

Geometry. All e.s.d.'s (except the e.s.d. in the dihedral angle between two l.s. planes)are estimated using the full covariance matrix. The cell e.s.d.'s are takeninto account individually in the estimation of e.s.d.'s in distances, anglesand torsion angles; correlations between e.s.d.'s in cell parameters are onlyused when they are defined by crystal symmetry. An approximate (isotropic)treatment of cell e.s.d.'s is used for estimating e.s.d.'s involving l.s. planes.
Refinement. Refinement of *F*^2^ against ALL reflections. The weighted *R*-factor *wR* andgoodness of fit *S* are based on *F*^2^, conventional *R*-factors *R* are basedon *F*, with *F* set to zero for negative *F*^2^. The threshold expression of*F*^2^ > σ(*F*^2^) is used only for calculating *R*-factors(gt) *etc*. and isnot relevant to the choice of reflections for refinement. *R*-factors basedon *F*^2^ are statistically about twice as large as those based on *F*, and *R*-factors based on ALL data will be even larger.

### Fractional atomic coordinates and isotropic or equivalent isotropic displacement parameters (Å^2^)

**Table:**

	*x*	*y*	*z*	*U*_iso_*/*U*_eq_	
C1	0.7042 (3)	0.8893 (2)	0.44458 (8)	0.0374 (4)	
C2	0.5046 (3)	0.8128 (2)	0.40196 (8)	0.0376 (4)	
C3	0.4934 (3)	0.84333 (19)	0.33033 (8)	0.0372 (4)	
H3	0.3631	0.7959	0.3031	0.045*	
C4	0.8652 (3)	1.01190 (19)	0.33360 (8)	0.0368 (4)	
C5	0.3180 (4)	0.7061 (2)	0.43522 (10)	0.0510 (5)	
H5	0.3435	0.6787	0.4833	0.061*	
C6	0.6377 (3)	0.9724 (2)	0.21870 (8)	0.0432 (4)	
H6A	0.4685	0.9390	0.2017	0.052*	
H6B	0.6512	1.0851	0.2110	0.052*	
C7	0.8363 (4)	0.8923 (2)	0.17686 (9)	0.0464 (5)	
C8	0.9931 (4)	0.8282 (3)	0.14242 (11)	0.0612 (6)	
H8	1.1175	0.7773	0.1151	0.073*	
N1	0.6616 (2)	0.93903 (17)	0.29646 (7)	0.0370 (4)	
N2	0.8664 (3)	0.98582 (16)	0.40649 (7)	0.0398 (4)	
H2	0.9818	1.0356	0.4312	0.048*	
O1	0.7344 (2)	0.87340 (16)	0.51012 (6)	0.0490 (4)	
O2	1.0262 (2)	1.09061 (15)	0.30334 (6)	0.0472 (4)	
O3	0.1324 (3)	0.65141 (19)	0.40376 (8)	0.0685 (5)	

### Atomic displacement parameters (Å^2^)

**Table:**

	*U*^11^	*U*^22^	*U*^33^	*U*^12^	*U*^13^	*U*^23^
C1	0.0360 (8)	0.0454 (9)	0.0308 (8)	0.0027 (7)	−0.0031 (6)	−0.0023 (7)
C2	0.0353 (8)	0.0442 (9)	0.0333 (8)	0.0011 (7)	−0.0018 (6)	−0.0066 (7)
C3	0.0326 (8)	0.0439 (9)	0.0350 (8)	0.0021 (7)	−0.0041 (6)	−0.0089 (7)
C4	0.0365 (8)	0.0414 (9)	0.0323 (8)	0.0027 (7)	−0.0036 (7)	−0.0023 (7)
C5	0.0503 (10)	0.0600 (11)	0.0427 (10)	−0.0103 (9)	−0.0011 (8)	−0.0042 (8)
C6	0.0451 (10)	0.0551 (10)	0.0294 (8)	0.0008 (8)	−0.0085 (7)	0.0013 (7)
C7	0.0533 (11)	0.0548 (11)	0.0311 (8)	−0.0078 (9)	−0.0027 (8)	−0.0019 (8)
C8	0.0625 (13)	0.0738 (14)	0.0474 (11)	−0.0033 (11)	0.0076 (10)	−0.0125 (10)
N1	0.0366 (7)	0.0471 (8)	0.0272 (7)	0.0016 (6)	−0.0044 (5)	−0.0029 (6)
N2	0.0402 (8)	0.0494 (8)	0.0297 (7)	−0.0079 (6)	−0.0084 (5)	−0.0014 (6)
O1	0.0500 (7)	0.0683 (8)	0.0285 (6)	−0.0120 (6)	−0.0057 (5)	0.0017 (5)
O2	0.0470 (7)	0.0566 (8)	0.0378 (7)	−0.0094 (6)	−0.0019 (5)	0.0039 (5)
O3	0.0592 (9)	0.0805 (11)	0.0658 (10)	−0.0216 (7)	0.0004 (7)	−0.0139 (8)

### Geometric parameters (Å, °)

**Table:**

C1—O1	1.2325 (19)	C5—O3	1.211 (2)
C1—N2	1.374 (2)	C5—H5	0.9300
C1—C2	1.449 (2)	C6—C7	1.462 (3)
C2—C3	1.356 (2)	C6—N1	1.475 (2)
C2—C5	1.465 (3)	C6—H6A	0.9700
C3—N1	1.351 (2)	C6—H6B	0.9700
C3—H3	0.9300	C7—C8	1.173 (3)
C4—O2	1.211 (2)	C8—H8	0.9300
C4—N2	1.371 (2)	N2—H2	0.8600
C4—N1	1.397 (2)		
			
O1—C1—N2	120.11 (14)	C7—C6—N1	112.24 (14)
O1—C1—C2	124.91 (15)	C7—C6—H6A	109.2
N2—C1—C2	114.98 (13)	N1—C6—H6A	109.2
C3—C2—C1	118.22 (15)	C7—C6—H6B	109.2
C3—C2—C5	120.68 (15)	N1—C6—H6B	109.2
C1—C2—C5	121.10 (15)	H6A—C6—H6B	107.9
N1—C3—C2	123.42 (14)	C8—C7—C6	178.99 (19)
N1—C3—H3	118.3	C7—C8—H8	180.0
C2—C3—H3	118.3	C3—N1—C4	121.52 (13)
O2—C4—N2	123.44 (14)	C3—N1—C6	121.56 (13)
O2—C4—N1	122.30 (14)	C4—N1—C6	116.91 (14)
N2—C4—N1	114.26 (14)	C4—N2—C1	127.43 (13)
O3—C5—C2	123.80 (18)	C4—N2—H2	116.3
O3—C5—H5	118.1	C1—N2—H2	116.3
C2—C5—H5	118.1		
			
O1—C1—C2—C3	179.16 (16)	O2—C4—N1—C3	175.63 (15)
N2—C1—C2—C3	−0.7 (2)	N2—C4—N1—C3	−4.1 (2)
O1—C1—C2—C5	−0.4 (3)	O2—C4—N1—C6	−4.8 (2)
N2—C1—C2—C5	179.81 (15)	N2—C4—N1—C6	175.44 (14)
C1—C2—C3—N1	1.3 (2)	C7—C6—N1—C3	−106.92 (18)
C5—C2—C3—N1	−179.19 (15)	C7—C6—N1—C4	73.53 (19)
C3—C2—C5—O3	−7.1 (3)	O2—C4—N2—C1	−174.72 (16)
C1—C2—C5—O3	172.38 (18)	N1—C4—N2—C1	5.0 (2)
C2—C3—N1—C4	1.3 (2)	O1—C1—N2—C4	177.47 (15)
C2—C3—N1—C6	−178.27 (15)	C2—C1—N2—C4	−2.7 (2)

### Hydrogen-bond geometry (Å, °)

**Table:**

*D*—H···*A*	*D*—H	H···*A*	*D*···*A*	*D*—H···*A*
N2—H2···O1^i^	0.86	1.98	2.8329 (18)	174.

Symmetry codes: (i) −*x*+2, −*y*+2, −*z*+1.
